# Closed, pumpless microphysiological system with unidirectional flow for co-culture and focal irradiation

**DOI:** 10.1039/d5ra06702b

**Published:** 2026-02-25

**Authors:** Hidetaka Ueno, Kenji Hanamura, Yuri Aoki, Mai Yamamura, Tomoaki Shirao, Takaaki Suzuki

**Affiliations:** a Center for Advanced Medical Engineering Research & Development (CAMED), Kobe University 1-5-1 Minatojima-minamimachi, Chuo-ku Kobe-City Hyogo 650-0047 Japan; b Department of Medical Device Engineering, Graduate School of Medicine, Kobe University 7-5-1 Kusunoki-cho Chuo-ku Kobe-City Hyogo 650-0017 Japan; c Health and Medical Research Institute, National Institute of Advanced Industrial Science and Technology (AIST) 2217-14 Hayashi-cho Takamatsu-City Kagawa 761-0395 Japan; d Department of Radiological Technology, Niigata University of Health and Welfare 1398 Shimami-cho, Kita-ku Niigata-City Niigata 950-3198 Japan kenji-hanamura@nuhw.ac.jp; e Department of Pharmacology, Gunma University Graduate School of Medicine 3-39-22 Showa-machi Maebashi-City Gunma 371-8511 Japan; f Division of Mechanical Science and Technology, Gunma University Graduate School of Science and Technology 1-5-1 Tenjin-cho Kiryu-City Gunma 376-8515 Japan; g AlzMed,Inc. 7-3-1, Hongo Bunkyo-ku Tokyo 113-8485 Japan; h Division of Mechanical Science and Technology, Gunma University 1-5-1 Tenjin-cho Kiryu-City Gunma 376-8515 Japan suzuki.taka@gunma-u.ac.jp

## Abstract

Intercellular communication is important for biological phenomena such as radiation-induced bystander effects (RIBEs). Microphysiological systems (MPSs), in which multiple groups of cells are co-cultured in a circulatory system, have been used to study complex intercellular communication. Because they can provide models that closely resemble the human body, human cells have been used in MPSs to replicate it. For recapitulating the intercellular communication in human bodies, experiments using cells differentiated from human induced pluripotent stem cells or human primary cells should be performed; however, they are expensive for a limited cell number. Therefore, MPSs should be scaled down as much as possible to analyze secreted substances with a minimum number of cells. In this study, we propose a closed, pumpless MPS (CPMS) that allows focal irradiation of X-rays to a part of minimized culture space. The CPMS was designed to operate with a minimum of approximately 200 µL of medium by isolating the culture space from the external space to prevent its evaporation. For the efficient circulation of the substances secreted from cells, a gravity-driven passive unidirectional flow was generated using water head pressure with a maximum flow rate of approximately 15.7 µL min^−1^. In the CPMS, cultured neurons survived for 21 d in a static condition and formed synapses. Even under unidirectional flow for 7 d, cultured neurons extended neurites and formed branches. Furthermore, focal irradiation of X-rays induced apoptosis of the hippocampal cells in the irradiated chamber. These results suggest that the CPMS will be useful for analyzing intercellular communication, such as secreted substance-mediated RIBEs. The CPMS is suitable for analyzing small quantities of rare and/or expensive cells, such as commercially available human cells, because of its compactness.

## Abstract 2

A microfluidic platform that enables co-culture and focal irradiation under closed microenvironments, providing a robust approach for analyzing intercellular communication.

## Graphical abstract

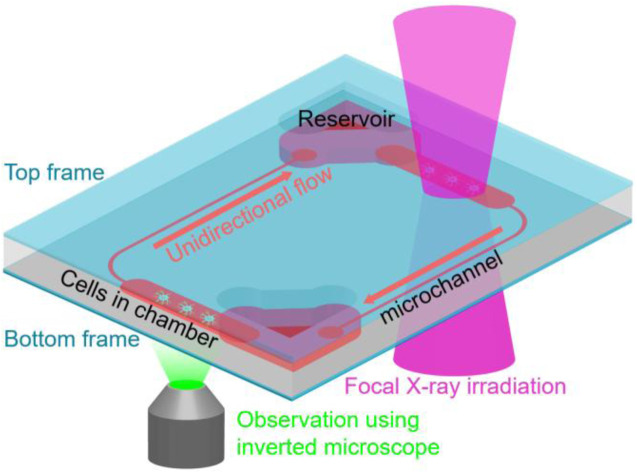

## Introduction

Radiotherapy is a common treatment for brain tumors; however, it can also affect normal neurons in the irradiated area. Recent clinical studies have demonstrated that hippocampal avoidance during whole-brain radiation therapy for brain metastases can better preserve the cognitive function of memantine-treated patients.^1–4^ However, whole-brain radiation therapy combined with memantine with hippocampal avoidance still induces cognitive failure.^5^ Therefore, understanding the mechanisms underlying the radiation-induced hippocampal dysfunction is crucial.

Damage to cells by ionizing radiation or ultraviolet (UV) radiation can affect cells that are not directly irradiated. This phenomenon is known as the radiation-induced bystander effect (RIBE) and is important for understanding the mechanisms of these radiation effects on cells or organs.^6–9^ For example, the RIBE is associated with the side effects of radiotherapy and the spread of UV light-induced photoreceptor cell death.^10,11^ Thus understanding the mechanisms of RIBE will be useful for reducing the side effects of radiotherapy and the spread of UV light-induced photoreceptor cell death.

RIBEs are mediated by direct intercellular communication using gap junctions or substances secreted from irradiated cells. Among the RIBEs, direct intercellular communication has been initially studied using films such as Mylar base dishes.^6,12,13^ Recently, microphysiological systems (MPSs), which are fabricated with high precision using semiconductor manufacturing technology, have been used for more accurate analysis to elucidate direct intercellular communication.^10,14–16^ Meanwhile, although analysis of RIBE mediated by substances secreted from irradiated cells has been performed by transferring cell culture inserts or medium containing secreted substances to another culture system,^17^ this process makes it difficult to analyze the indirect mechanisms mediated by substances secreted and working immediately after irradiation because it takes time. Therefore, the RIBEs induced by indirect mechanisms mediated by secreted substances have not been well studied. Thus, new MPSs that can efficiently reproduce intercellular communication mediated by secreted substances are required. In addition, for recapitulating the intercellular communication in human bodies, experiments using cells differentiated from human induced pluripotent stem (iPS) cells or human primary cells should be performed; however, they are expensive for a limited cell number. Therefore, MPSs should be scaled down as much as possible to analyze secreted substances with a minimum number of cells.

To understand the effect of secreted substances, previous research on MPSs has proposed a device that minimizes the amount of culture medium using pumpless systems.^18,19^ The authors suggest that reducing the medium volume enables drug and metabolite concentrations to more closely resemble those *in vivo*, which enhances the predictive accuracy of toxicity and pharmacological efficacy assessments. However, the system with a bidirectional flow used in previous research is not suitable for stable and efficient metabolite circulation compared to a system with a unidirectional flow because the direction of shear stress is not constant. In contrast, a gravity-driven unidirectional flow system requires a set distance between the inlet and outlet to generate a hydraulic head pressure difference. This approach enables simplified handling and operation; however, since it increases the volume of the culture space, the number of cells and volume of culture medium required for the assay are also increased.^20–22^ In addition, since the concentration of metabolites in the culture medium changes according to the evaporation of the culture medium, evaporation of the culture medium must be considered when minimizing the volume of the culture medium.^23^ Since multiple factors are involved, it remains challenging to develop a platform or system that can accurately recapitulate cell–cell interactions due to secreted substances produced from stimulated cells by external stimuli, such as irradiation with X-rays. Although individual aspects, such as medium volume, evaporation, and local stimulation, have been investigated separately in several previous studies, no study has yet comprehensively optimized these factors within a single platform.

We propose a closed, pumpless MPS (CPMS) capable of culturing neurons with a minimal amount of culture medium. The culture space in the CPMS is isolated from the outside by glass substrates to prevent the evaporation of the culture medium. The CPMS consists of two chambers for cell culture and microchannels connecting the two chambers. In our previous preliminary study, CPMS can generate unidirectional flow and culture cell line cancer cells in the chamber.^24^ In this study, we evaluated the usefulness of CPMS as the MPS for co-culturing and stimulating rare cells, such as primary cells, by conducting focal irradiation of X-rays to cultured neurons. Neurons were introduced into each chamber. The culture medium was efficiently perfused between the two chambers using unidirectional flow. The proposed CPMS, in which the culture medium circulates on a thin glass substrate, enables observation of cultured cells by inverted microscopy and focal irradiation of X-rays to the cultured cells. In this study, the functions of the CPMS for focal irradiation in an isolated culture space from the outside of the CPMS were evaluated.

The CPMS was fabricated by soft lithography using polydimethylsiloxane (PDMS), which is generally used for microdevices for cell culture. To evaluate the effect of culture medium evaporation on cells, we operated CPMS with its reservoirs open or closed with a top frame made of thin glass and observed the growth of cultured neurons. In addition, the effect of the unidirectional flow in the CPMS on the cultured neurons was also evaluated. Furthermore, the function of focal irradiation of X-rays in the proposed system was examined by observation of the cultured neurons when X-rays were irradiated to one of the chambers with neurons.

## Material and methods

### Closed and pumpless design approach for focal irradiation

**Fig. 1:**
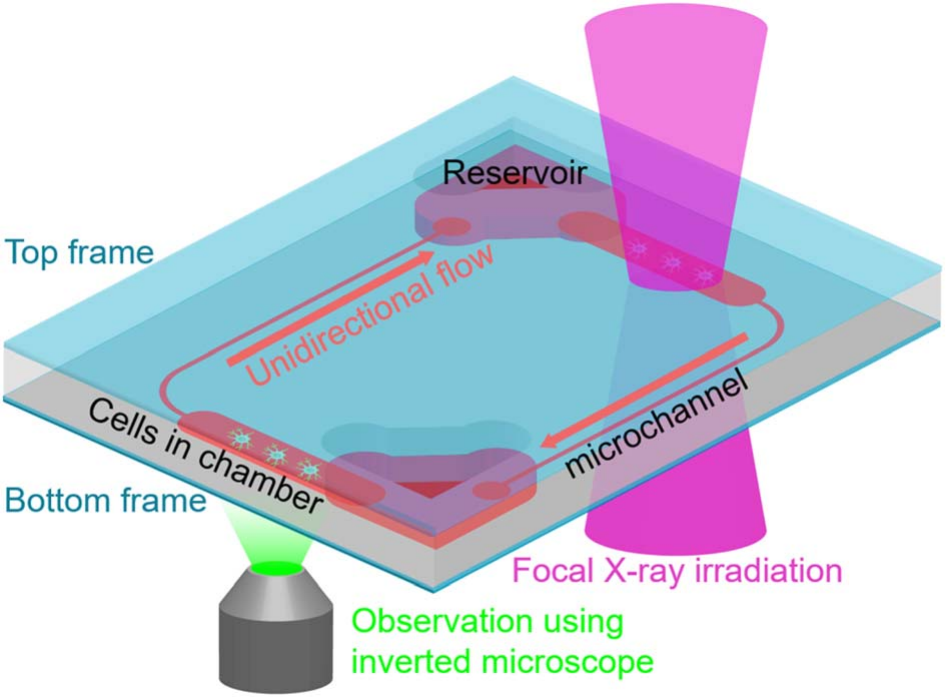
Closed, pumpless microphysiological system (CPMS). The CPMS was designed for isolating the space for cultured cells and circulating culture medium from the outside by sealing it with top and bottom thin glass frames. The culture medium was circulated unidirectionally by a gravity-driven passive flow using the water head pressure generated by tilting the proposed system. Since no driving mechanisms, such as pumps or active valves, are used in the isolated space, the excess space is minimized. In this system, cells were cultured on a bottom-thin glass frame. It allowed observation of the cells and focal irradiation of X-rays without exposing the culture medium to the outside.

The culture space containing the culture medium and the air in the reservoir within the proposed CPMS was designed to be isolated from the outside of the CPMS. By isolating from the outside of the CPMS, the evaporation and volume changes of the culture medium in the chambers, reservoirs, microchannels, and airflow pathways were prevented. By preventing evaporation of the culture medium, a cell culture system can culture a smaller number of cells using a smaller amount of culture medium than those used in previous studies, enabling reactions between cells using small amounts of cell-produced substances. A gravity-driven unidirectional flow mechanism using water head pressure allowed the culture medium to passively perfuse in one direction in a closed space. In unidirectional flow, the medium perfuses in one direction, generating not only one-directional shear stress but also transporting substances generated by irradiated cells to unirradiated cells in other cell culture chambers more efficiently compared to bidirectional flow. Additionally, the unidirectional flow was suitable for this closed system concept because it is generated without requiring driving mechanisms such as pumps and active microvalves. Thin glass substrates with high transparency and low autofluorescence used for the top and bottom frames could isolate the inside of the CPMS from the outside. Using a thin glass substrate, the focal irradiation of X-rays can be conducted efficiently because the thin glass substrate minimally absorbs X-rays. Meanwhile, MPSs for evaluating RIBEs require the ability not only to carry out focal irradiation of X-rays to cultured cells in the MPS but also to observe the cells with a microscope. The use of these glass substrates enabled focal irradiation of X-rays and observation using an inverted microscope without exposing the culture medium to the outside (Fig. 1).

### Detailed design and FEM analysis

**Fig. 2:**
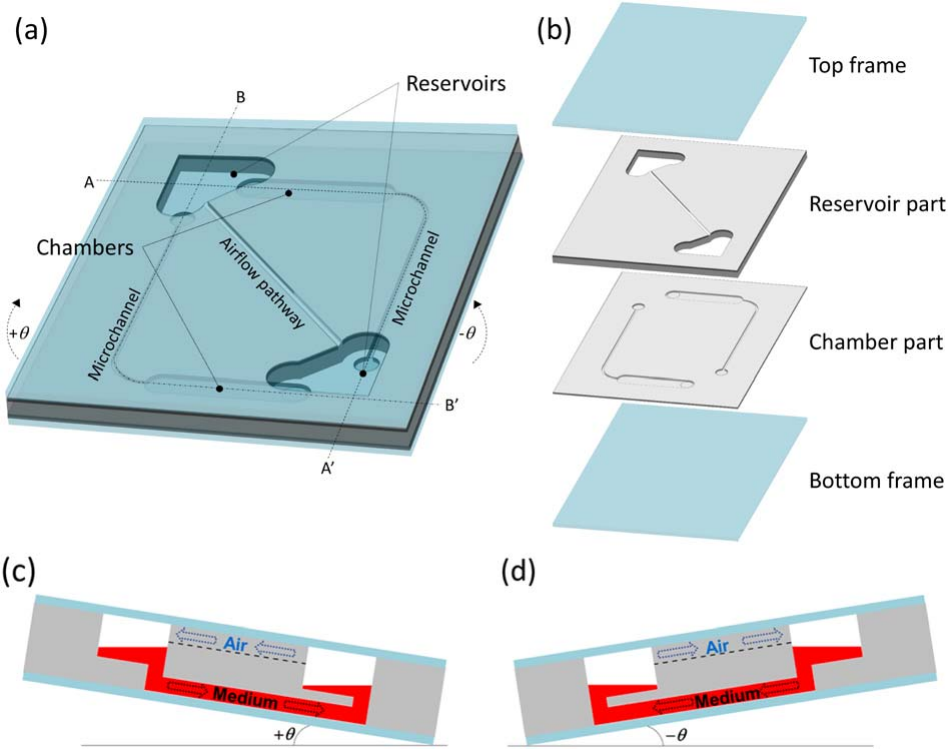
Schematic of the CPMS. (a) The CPMS had two reservoirs, two chambers, two microchannels, and one airflow pathway. (b) The four elements consist of the CPMS. (c) The culture medium flows between reservoirs through the chamber and microchannel shown in the A–A′ cross-section when the CPMS is tilted at +*θ*. (d) The cell culture medium flows between reservoirs through another chamber and another microchannel shown in the B–B′ cross-section when the CPMS is tilted at −*θ*. At the same time, the air in the reservoirs is exchanged by the airflow pathway connecting the two reservoirs directly.^24^

The CPMS had two cell culture chambers, two reservoirs, two microchannels, and an airflow pathway (Fig. 2(a)). Each chamber was connected to one microchannel. The two reservoirs were connected by an airflow pathway located in a higher position compared to the chambers.

The CPMS consisted of four elements: the top frame, reservoir part, chamber part, and bottom frame (Fig. 2(b)). The top frame and the bottom frame were made of thin glass substrates. The other part was made of a silicone material, PDMS. The reservoir part had two reservoirs and an airflow pathway connecting the two reservoirs. The chamber part had two chambers for cell culture, two microchannels, and four inlets/outlets.

The culture medium in the CPMS was circulated by the water head pressure difference generated by tilting the system using a rocking platform, which can cause the CPMS to tilt back and forth at constant time intervals. The air in the reservoirs was exchanged through the airflow pathway.

**Fig. 3:**
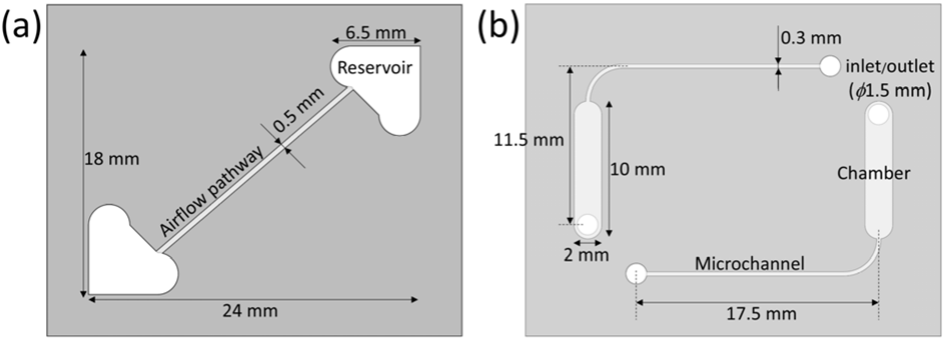
Schematics of elements made of PDMS in the CPMS. (a) The reservoir part with two triangle-shaped holes and an airflow pathway. (b) The chamber part with two chambers and two microchannels.

The two-dimensional schematic images of the reservoir part and chamber part are shown in Fig. 3. The reservoir part had two triangle-shaped holes working as reservoirs. The area of the holes was 30.1 mm^2^. These holes were connected by an airflow pathway for exchanging air between reservoirs directly. By exchanging air, the air pressure in both reservoirs was maintained constant even though the medium level in the reservoir was changed. Therefore, the unidirectional medium flow depended on only the difference in the medium level. The height and depth of the airflow pathway were 0.5 mm (Fig. 3(a)). The chamber part had two chambers for cell culture and two microchannels. These chambers and microchannels had holes, working as inlets and outlets, connecting to reservoirs. The length and width of the chambers were 10 mm and 2 mm, respectively. The width of the microchannel was 0.3 mm (Fig. 3(b)). By this design, this device can be operated using approximately 200 µL of medium. This volume is smaller than that used in most of the previous research using unidirectional flow.^20,22,25^ This characteristic is advantageous for culturing rare cells, such as iPS cell-derived cells or primary human cells, at low cost.

To evaluate the effect of shear stress on the culture cells, the shear stress to cells and flow rate were simulated by FEM analysis. The shear stress and flow rate depended on the pressure drop between the inlet and outlet and the size of the microchannel. The hydrostatic pressure drop between the inlet and outlet was calculated using eqn (1).

1 Δ*P* = *ρgH* (Pa),

where *ρ* is the density of the culture medium, *g* is the gravitational acceleration, and *H* is the difference in height of the liquid surface. The pressure difference Δ*P* was proportional to the height difference *H*. The hydraulic resistance of the chamber and microchannel *R* is calculated using eqn (2).

2 *R* = Δ*P*/*Q* ((Pa × s) per m^3^)

*Q* is the flow rate in the chamber and microchannel. *Q* is inversely proportional to the hydraulic resistance *R* when Δ*P* is constant. The hydraulic resistance of the chamber and microchannel *R* is also calculated using eqn (3).

3 *R* = [12*ηL*/(1 − 0.63(*h*/*w*))] × (1/*wh*^3^)

*η* is the kinematic viscosity. *L*, *w*, and *h* are the length, width, and height of the chamber and microchannel, respectively (*w* > *h*). Since the hydraulic resistance *R* in the chamber and microchannel is highly dependent on the height of the chamber and microchannel *h*, the flow rate and the shear stress to the cells were simulated on different *h* values.

**Fig. 4:**
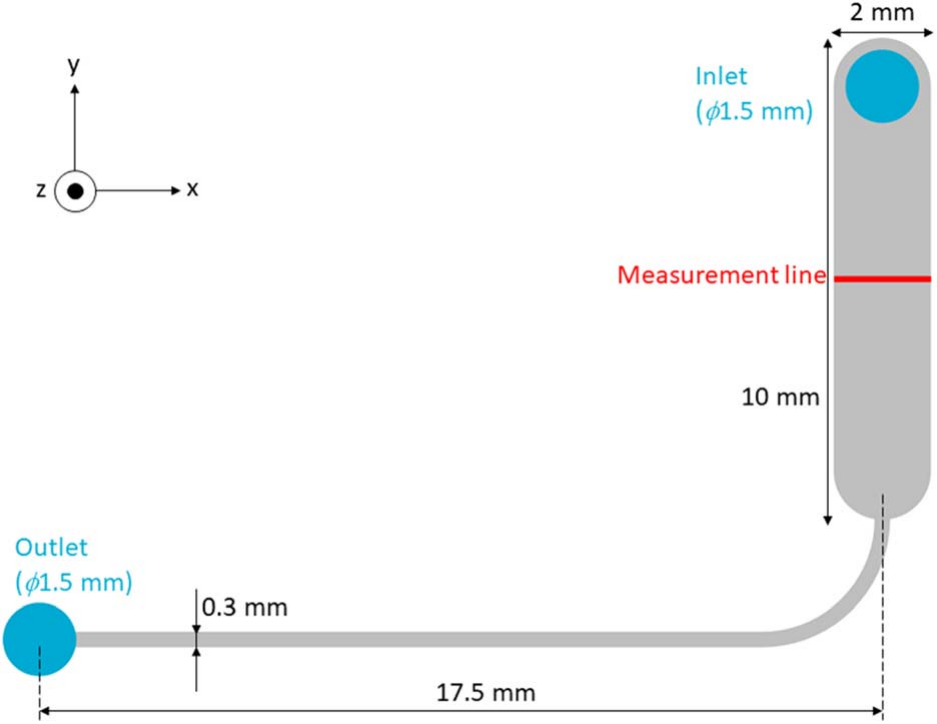
FEM analysis model. One of the chambers/microchannels in the chamber part was designed as the analysis model. The pressure was set at 21.582 Pa at the inlet and 0 Pa at the outlet. The height of the model *h* was changed from 20 to 500 µm. The shear stress and flow rate at the measurement line were calculated.^24^

In the proposed system, a medium flow was generated in the chamber/microchannel, and cells were cultured under the medium flow. The cells under the medium flow suffered from shear stress.^25,26^ In natural environments such as human and animal bodies, only vascular endothelial cells and lymphocytes suffer under shear stress. Normally, other cells, such as neurons, do not suffer under shear stress in not only human and animal bodies but also static culture conditions such as normal cell culture dishes. To elucidate and evaluate the flow rate of the culture medium and the shear stress applied to the cells in the chamber, FEM analysis was performed using static flow analysis of COMSOL analysis software (COMSOL Multiphysics 5.4; COMSOL, Inc.) with the height of the chamber and microchannel, *h*, as a parameter. The FEM model is shown in Fig. 4. The analytical model reproduced the chamber and microchannel. A pressure of 21.582 Pa was applied to the inlet, assuming a 2 mm height difference *H* in the liquid surface between the two reservoirs. The shear stress at 3 µm from the bottom of the chamber was calculated while changing the height of the chamber and microchannel *h* from 20 to 500 µm.

### Fabrication

The proposed CPMS consisted of two thin glass substrates, the reservoir part, and the chamber part. The reservoir and chamber parts were fabricated using soft lithography. As the first step of the reservoir part fabrication, the mold structure was fabricated using a 3D printer (Form2; Formlabs). A main agent and crosslinker of PDMS were mixed at a 9 : 1 volume ratio and heated at 80 °C for 2 h on the mold structure. After the polymerization reaction, PDMS was released from the mold structure. For the chamber part fabrication, the glass substrate (cover glass, 30 mm × 40 mm, no. 5; Matsunami Glass Ind., Ltd) was initially cleaned with piranha solution. The adhesion-promoting agent (OAP; Tokyo Ohka Kogyo Co., Ltd) was coated on the glass substrate using a spin coater (1H-DX2; Mikasa Co., Ltd) with a rotation speed of 4000 rpm. By heating at 200 °C for 1 min, the solvent was removed. Next, the negative photoresist (SU-8 3050, Nippon Kayaku Co., Ltd) was deposited using a spin coater (1H-DX2; Mikasa Co., Ltd) with a rotation speed of 500 rpm for 30 s. By heating at 65 °C for 5 min and 95 °C for 30 min, the solvent was removed. After cooling down to room temperature, the SU-8 layer was exposed with an energy of 1200 mJ cm^−2^. For post-exposure baking, the exposed SU-8 was heated at 65 °C for 5 min and 95 °C for 5 min. After cooling down to room temperature, the SU-8 layer was developed using a photoresist developer (SU-8 developer, KAYAKU Advanced Materials, Inc.). A main agent and crosslinker of the PDMS were mixed at a 9 : 1 volume ratio and were heated at 80 °C for 2 h on the SU-8 mold structure. After the polymerization reaction, the PDMS was released from the mold structure. The glass substrate and the chamber part were treated by O_2_ ashing (RIE-10NR; Samco Inc.) to activate and clean their surfaces. The treated glass substrate and chamber part were combined by attaching their surfaces and heating at 80 °C for 2 h. Finally, after combining the chamber part and glass substrate, the reservoir part adhered using uncured PDMS as glue. For curing PDMS, it was heated at 80 °C for 2 h.

The top frame, which serves as the cover glass and the reservoir part are brought into close contact by pressure applied by a jig. Since the upper surface of the reservoir part made of PDMS had cured in an open-air environment, it was smooth. This flat surface forms a tight seal with the equally flat surface of the top frame made of a cover glass, resulting in complete isolation from the external environment. On the other hand, it was capable of removing the top frame by releasing pressure by the jig easily.

### Evaluation of fabricated CPMS

The sizes of the fabricated chamber and microchannel were measured using a surface texture measuring instrument (Surfcom 130A; Tokyo Seimitsu Co., Ltd). The average and standard deviation were calculated from the measured size at three locations.

To measure the flow rate inside the CPMS, 206 µL of deionized (DI) water containing fluorescent beads (G1000; Thermo Fisher Scientific) was introduced into the CPMS. The CPMS was tilted by 9°, and the fluorescent beads in the microchannel were observed. For measuring the flow rate of the microchannel, the velocity of the fluorescent beads that flowed center of the microchannel was measured. The flow rate in the microchannel was calculated by taking half of the measured velocity of beads as the average flow velocity because the velocity of beads followed velocity profile under laminar flow. The substance generated by X-ray-irradiated cells should be transferred to cells cultured in another chamber by tilting the CPMS to mix the medium. To evaluate the mixing function of the CPMS, the reservoirs and chambers of CPMS were filled with 200 µL of DI water. Then, 6 µL of uranine solution (0.2 w/v% uranine solution; FUJIFILM Wako Chemicals Co., Ltd) was introduced into one of the reservoirs and chambers. The CPMS was tilted by 9° and allowed to stand for 30 s, then tilted by 9° in the opposite direction and allowed to stand for 30 s. Before and after repeating this operation five times, the fluorescence images were observed. The fluorescence intensity was measured, and the ratio between the two chambers was calculated.

The inverted fluorescence microscope (IX71; Evident) was used to observe the fluorescence of the fluorescent beads and uranine solution.

### Cell culture and immunocytochemistry

Animal experiments were performed according to the guidelines of the Animal Care and Experimentation Committee (Gunma University, Showa Campus, Maebashi, Japan) and conformed to NIH guidelines for the use of animals in research. Every effort was made to minimize animal suffering and to reduce the number of animals used. The hippocampal culture was performed using methods shown in previous research.^27,28^ In brief, frozen hippocampal cells prepared from the hippocampus of Wistar rats at embryonic day 18 (Charles River) were mixed with Neurobasal Medium containing B27 supplement, penicillin–streptomycin, and Glutamax. Subsequently, the mixture was centrifuged at 800 rpm for 5 min, and the supernatant was removed and resuspended in the same culture medium. Cells were seeded at a density of 3.0 × 10^4^ cells per cm^2^ into a chamber part previously coated with polylysine or onto 96-well plates (µ-plate 96 well, ibidi ib89626) and cultured in 206–360 µL of culture medium. Medium change was performed 2 h after seeding. Cultured hippocampal cells were fixed in 4% paraformaldehyde in 0.1 M PB for 20 min to 30 min. Cultured cells were permeabilized with 0.1% Triton X in PBS for 5 min. Then, the cells were incubated with 3% bovine serum albumin in PBS (PBSA) for 1 h and with primary antibodies containing *anti*-drebrin antibody (mouse monoclonal, clone M2F6, hybridoma supernatant, 1 : 1) to detect excitatory synapses and anti-MAP2 antibodies (rabbit polyclonal, AB5622, 1:2000, Merck Millipore Darmstadt, Germany) to detect neuronal cell bodies and dendrites.^27,29^ After washing three times with PBS, cells were treated with Alexa Fluor 488-conjugated donkey anti-mouse IgG (1:250, Jackson Immunoresearch) and Alexa Fluor 594-conjugated donkey anti-rabbit IgG (1:250, Jackson Immunoresearch) in PBSA for 2 h at room temperature. Finally, after washing with PBS, the cells were stored in PBS containing 0.1% sodium azide.

### Static and dynamic culture

The effect of isolating the cell culture space in the CPMS from the outside on cell growth was evaluated in a static cell culture. Hippocampal cells in the CPMS were incubated in the incubator for 21 d. As a control, hippocampal cells were cultured in the system without a top frame and in the 96-well plate. Hippocampal neurons incubated in the CPMS were observed 1 d and 21 d after seeding.

The effect of the dynamic culture environment with unidirectional medium flow on the growth of hippocampal neurons in the CPMS was evaluated. After hippocampal cells were introduced into the CPMS, the top frame was used to close the reservoir and prevent evaporation of the culture medium. For the comparison, cultured hippocampal cells in the system without the top frame were used. The CPMS was placed on a rocking mixer (NA-M101, Nissinrika) in the incubator. The rocking mixer made inclination angles of ±9° at constant intervals to generate unidirectional medium flow in the CPMS. After culturing hippocampal neurons for 7 d under both conditions, the hippocampal neurons were observed. The number of hippocampal neurons with neurites per area (0.147 mm^2^) was counted manually to evaluate the culture condition.

Phase contrast and fluorescence images of hippocampal neurons in the CPMS and the images in the 96-well plate were observed using an inverted microscope (IX81, Evident). For the phase contrast images in the CPMS and the images in 96-well plates, a 20× 0.50 numerical aperture objective lens was used. Fluorescence images in the CPMS were observed using a 60× 1.42 numerical aperture objective lens. The hippocampal cell number and the neuron number with neurites were quantified using Fiji (ImageJ, https://imagej.net/software/fiji/). The dendrite length was estimated from DAPI and MAP2 images. These images were thresholded using the “moments” thresholding modules and binarized. Binarized MAP2 images were shrunk and multiplied by binarized DAPI images using “image calculator” module to detect neuronal cell bodies. The size of the neuronal cell bodies was recovered using a maximum filter. To create dendritic images, neuronal cell body signals were subtracted from binarized MAP2 images *via* “omage calculator” module. The “Skeletonize (2D/3D)” plugin was used to visualize the dendritic skeleton. The length was then estimated by measuring the number of the pixels of the skeleton. Before detecting drebrin clusters, background values were removed from the images. The linear density of drebrin clusters was defined as regions with a peak fluorescent intensity at least two-fold greater than the averaged fluorescence intensity of the dendrites, using the threshold function of Metamorph software (Molecular devices).

### Focal irradiation of X-rays to establish an experimental model for detecting RIBE

To reproduce RIBEs in the CPMS, one of the chambers was irradiated by a focal X-ray. Hippocampal cells were introduced into CPMS and cultured for 24 h. Hippocampal cells were observed using an inverted microscope (IX81, Evident) with a 20× objective lens. The cultured hippocampal cells were irradiated with 2 Gy X-rays in one of the chambers (1.3 Gy min^−1^, Shimadzu X-TITTAN 225S X-ray generator, Shimadzu Inc.). The areas other than the irradiated chamber were shielded from radiation exposure with lead plates. Hippocampal cells were observed 6 h after irradiation. Effects of focal irradiation on cultured hippocampal neurons were examined by observation of the cells in the shielded non-irradiated chamber and irradiated chamber. Apoptosis of hippocampal cells was evaluated by the presence of pyknotic nuclei in the phase contrast images.^30^

## Results

### System design and operation

**Fig. 5:**
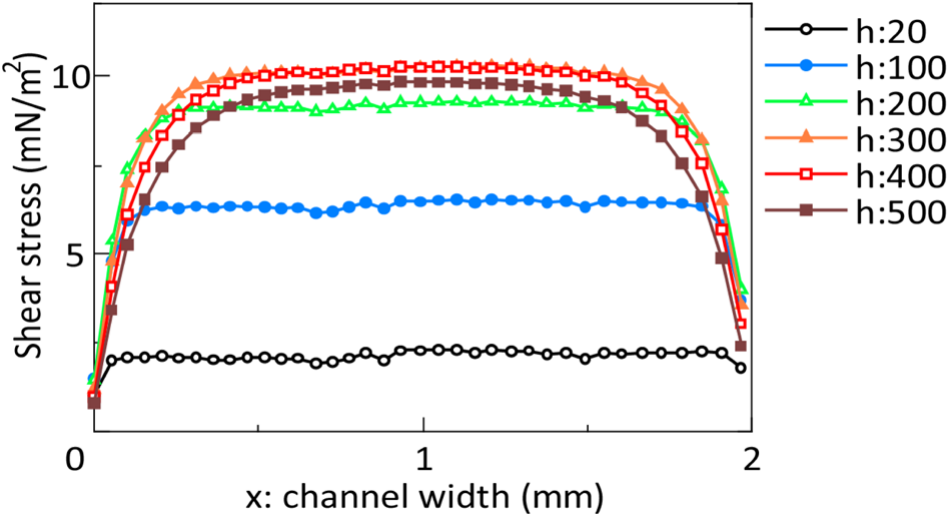
Distribution of shear stress in the *x*-axis direction of the chamber. The chamber/microchannel height *h* is 20 µm and measured from 100 to 500 µm at intervals of 100 µm.

The flow rate and shear stress of the cultured cells were simulated by FEM analysis. The shear stress at measurement lines at different heights is shown in Fig. 5. Shear stresses are shown for each chamber/microchannel height *h* at the measurement line, 3 µm in the height direction from the chamber bottom. The ends of the horizontal axis are the side walls of the chamber. The vertical axis indicates the simulated shear stress. The simulated results, which were obtained using a simulation model with chamber/microchannel height *h* ranging from 20 µm to 500 µm, are plotted. The shear stress had a maximum value at chamber/microchannel heights *h* of 300 to 400 µm. The shear stress decreased near the side walls of the chamber. The shear stress remained almost constant regardless of the chamber/microchannel height *h* from 500 to 1500 µm. It means, that shear stress was spatially uniform across the central region of the channel width, independent of channel height.

**Fig. 6:**
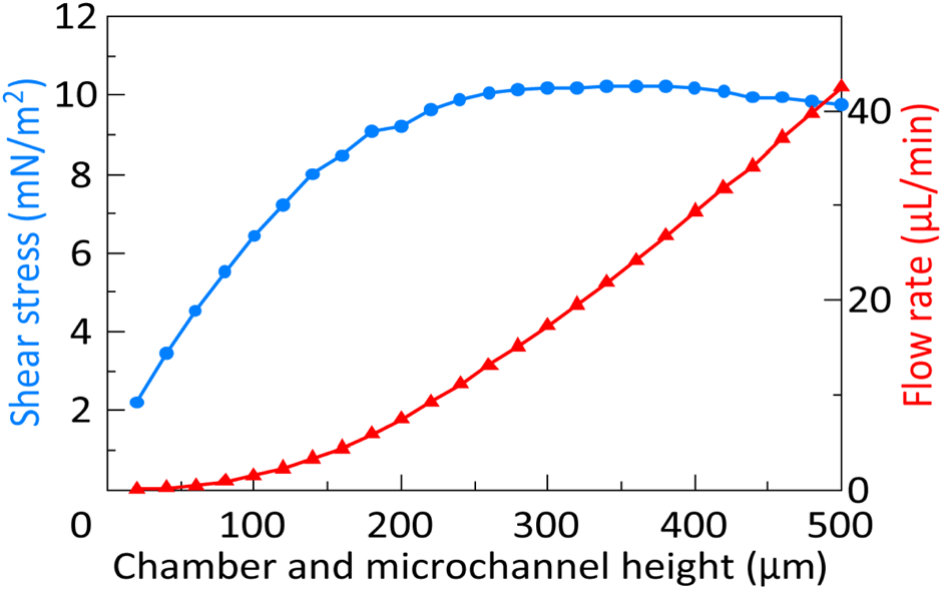
Shear stress and flow rate at different chamber/microchannel heights.^24^

The average shear stress and flow rate at 800 to 1200 µm in the *x*-axis direction of the chamber were calculated. The simulated shear stress and flow rate are shown in Fig. 6. The *x*-, *y*-, and *r*-axes indicate the chamber/microchannel height *h*, average shear stress, and flow rate, respectively. The shear stress increased to the chamber/microchannel height *h* up to approximately 300 µm, reached a maximum at a chamber/microchannel height *h* of 300 to 400 µm, then decreased at a chamber/microchannel height *h* of over 400 µm. The flow rate constantly increased to the chamber/microchannel height *h* up to 500 µm.

The sizes of the fabricated chamber and microchannel made of PDMS were measured using the surface texture measuring instrument (Surfcom 130A; Tokyo Seimitsu Co., Ltd). The height of the chamber and microchannel of CPMS was 404.4 ± 22.9 µm.

**Fig. 7:**
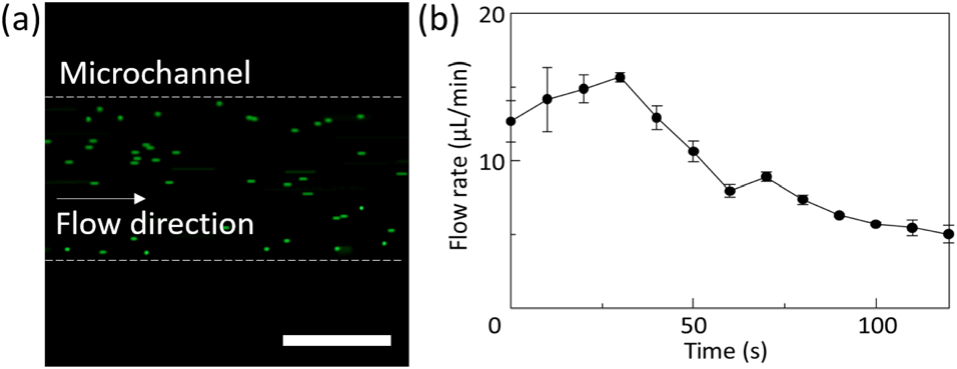
Measurement of flow rate in the microchannel. (a) Fluorescent image of fluorescent beads flowing in a microchannel. The scale bar indicates 200 µm. (b) The changes in the flow rate in the microchannel after tilting the CPMS.^24^

The flow rate in the chamber and microchannel was calculated by measuring the movement of fluorescent beads after tilting the CPMS. The fluorescent image and the calculated flow rate are shown in Fig. 7. The fluorescent beads flowed in one direction. The average flow rate ranged from 5 to 15 µL min^−1^ and decreased over time.

**Fig. 8:**
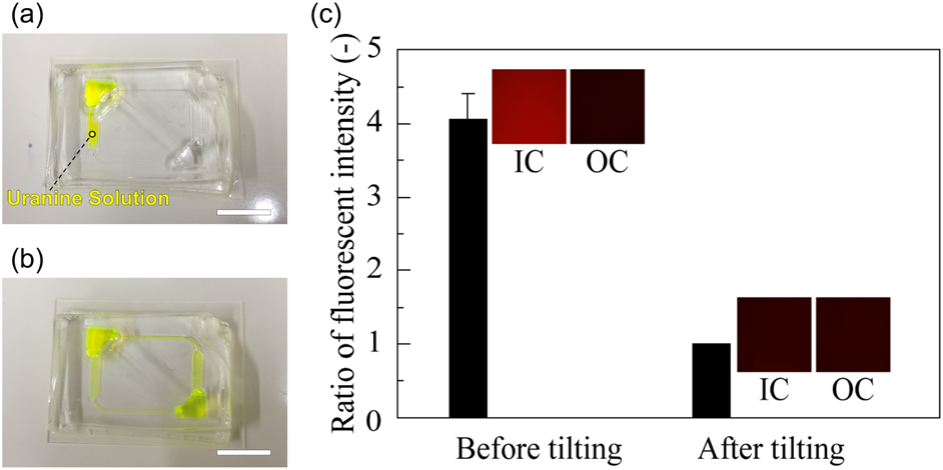
Changes in the fluorescent intensity in the chamber before and after tilting the CPMS. (a) Photograph of CPMS immediately after introducing the uranine solution. The scale bar indicates 10 mm. (b) The photograph of the CPMS after tilting five times. (c) Ratio of fluorescent intensity between chambers before and after tilting the CPMS. Fluorescence images, which are the uranine-solution-induced cell culture chamber (IC) and other cell culture chamber (OC), are also shown in the graph.

The mixing function of CPMS was evaluated using a uranine solution. The images of CPMS and graphs of fluorescence intensity ratio before and after tilting are shown in Fig. 8. Immediately after the addition of uranine solution to one of the reservoirs and chambers, the uranine solution was observed only in one chamber, and the fluorescent intensity was four times higher compared to that of another chamber (Fig. 8(a) and (c)). After tilting five times, the uranine solution was observed in all chambers, microchannels, and reservoirs. The fluorescent intensity was almost equal between the two chambers (Fig. 8(b) and (c)).

### System performance: static culture

**Fig. 9:**
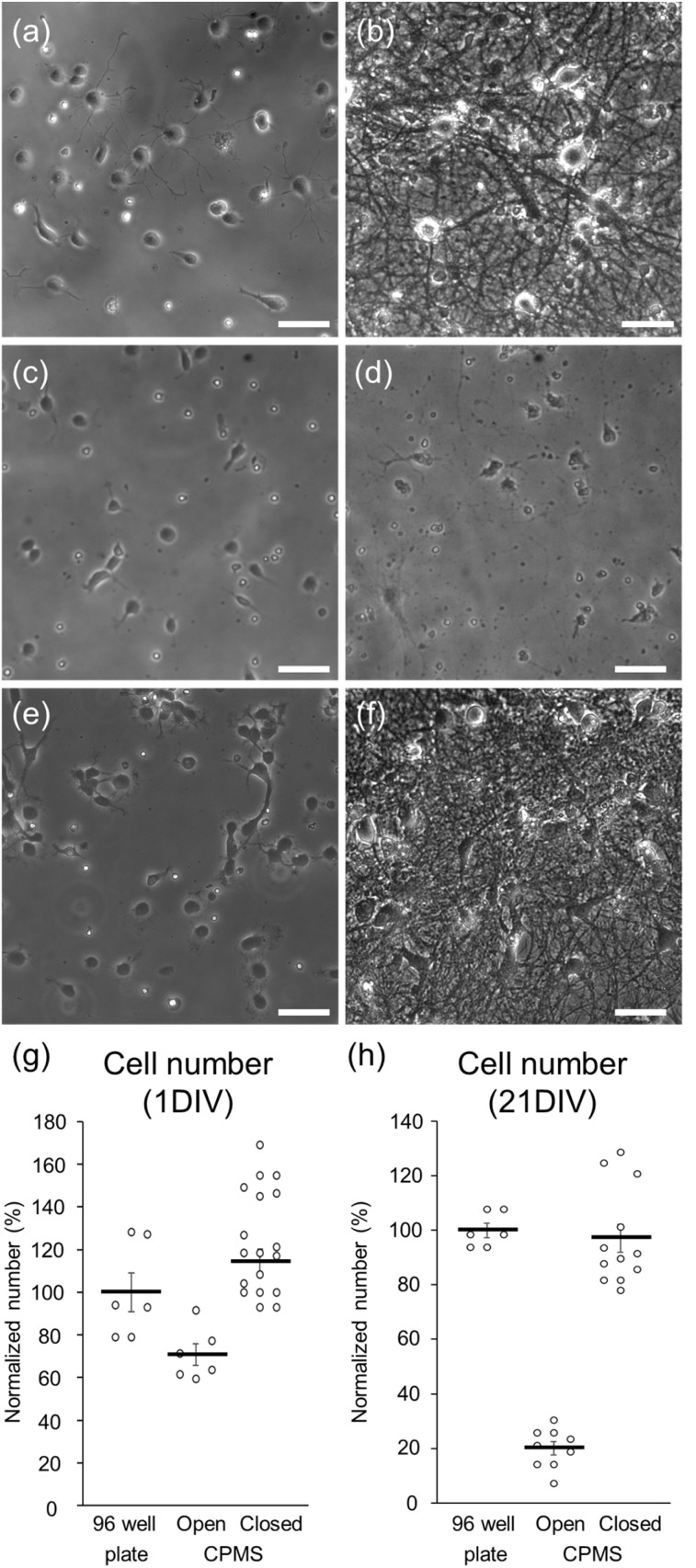
Phase contrast images of hippocampal neurons. (a, c and e) Cultured hippocampal neurons 1 d after seeding in a 96-well plate (a), in an open chamber (c), and in a closed chamber (e). (b, d and f) Cultured hippocampal neurons 21 d after seeding in a 96-well plate (b), in an open chamber (d), and in a closed chamber (f). (g and h) Number of hippocampal cells 1 d after seeding (g) and 21 d after seeding (h) normalized with average cell number in 96-well plates. Data were presented as mean ± S.E.M.

**Fig. 10:**
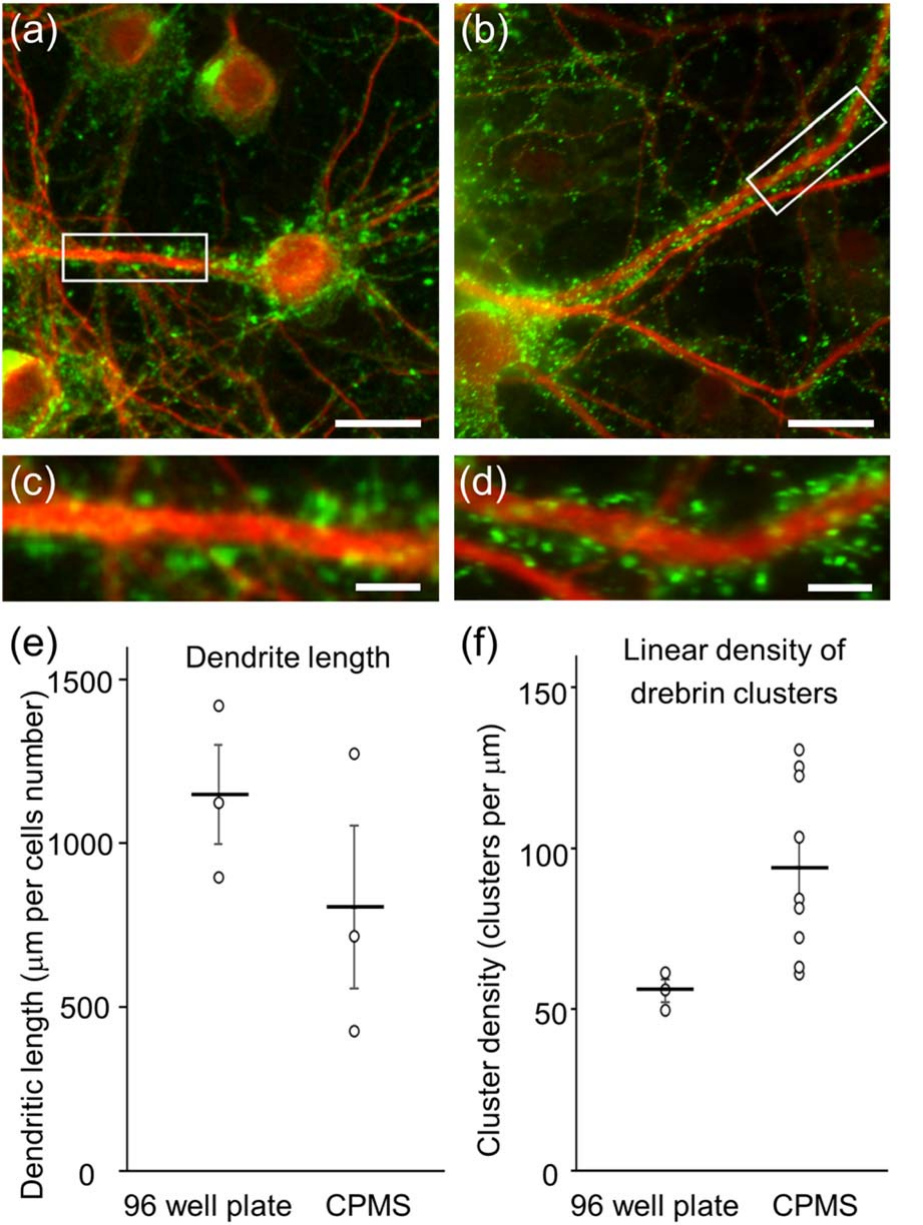
Fluorescent images of cultured hippocampal neurons 21 d after seeding. Red: MAP2. Green: drebrin. (a) Hippocampal neurons in the 96-well plate. (b) Hippocampal neurons in the CPMS with a closed reservoir. (c) Enlarged image of hippocampal neurons in the 96-well plate. (d) Enlarged image of hippocampal neurons in the CPMS with a closed reservoir. The scale bars indicate 20 µm in (a) and (b), and 5 µm in (c) and (d). (e) Quantitative analysis of dendrite length per neuron. (f) Quantitative analysis of linear density of drebrin clusters along dendrites. Data were presented as mean ± S.E.M.

After seeding hippocampal neurons in the chamber part of the CPMS, hippocampal neurons were cultured with two conditions, in which the reservoir in the CPMS was closed (sealed) or open. The hippocampal neurons were also cultured in 96-well plates as described in previous studies.^27,28^ In 96-well plates, the neurons extended several long neurites and showed the accumulation of an actin-binding protein, drebrin, along dendrites (Fig. 9(a, b, g, h) and 10(a, c, e, f)). In contrast, although a lot of neurons cultured in the CPMS began to grow protrusions on the first day of culture, few protrusions were observed after 21 d of culture in the CPMS with an open reservoir (Fig. 9(c and d)). Hippocampal neurons cultured in the CPMS with a closed reservoir could extend fine dendrites and exhibited accumulation of drebrin along dendrites 21 d after seeding (Fig. 9(e, f) and 10(b, d, e, f)), indicating proper synapse formation.^27^

### System performance: dynamic culture

**Fig. 11:**
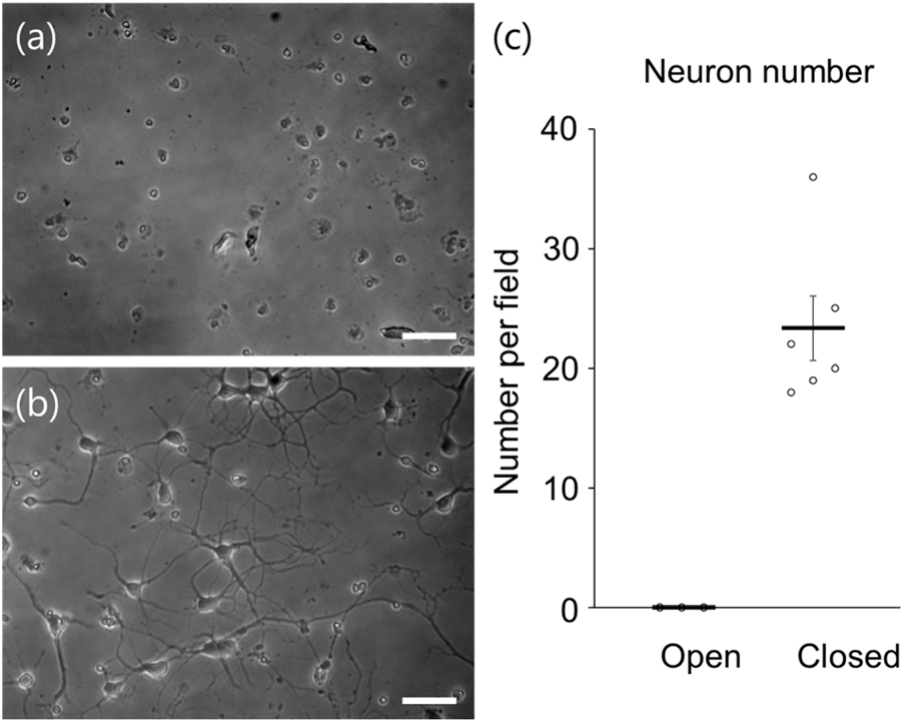
Hippocampal neurons cultured under unidirectional flow for 7 d. (a) In the CPMS with open reservoirs, there were no neurons with neurites 7 d after seeding. (b) In the CPMS with closed reservoirs, neurons showed extensive neurite outgrowth. The scale bars indicate 50 µm. (c) Number of hippocampal neurons with neurites per field in the CPMS with open or closed reservoirs under unidirectional flow for 7 d. These data were quantified from a representative experiment, and the imaging fields were randomly selected from areas that are not close the edges of the chambers. Data were presented as mean ± S.E.M.

The CPMSs were placed on a rocking mixer tilted at a 9° angle of inclination, and the rocking mixer was placed in the incubator. Hippocampal neurons were cultured under perfusion for 7 d after seeding hippocampal cells to the CPMS with closed reservoirs. For comparison, hippocampal neurons were cultured in the CPMS with open reservoirs. In the CPMS with open reservoirs, most hippocampal neurons have pyknotic nuclei and do not have neurites (Fig. 11(a)). On the other hand, hippocampal neurons in the CPMS with closed reservoirs extended their neurites (Fig. 11(b)). The number of neurons with extended neurites in each image was 0 ± 0 per area in the CPMS with open reservoirs and 23.3 ± 2.7 per field in the CPMS with closed reservoirs (Fig. 11(c)).

### Focal irradiation of X-rays

**Fig. 12:**
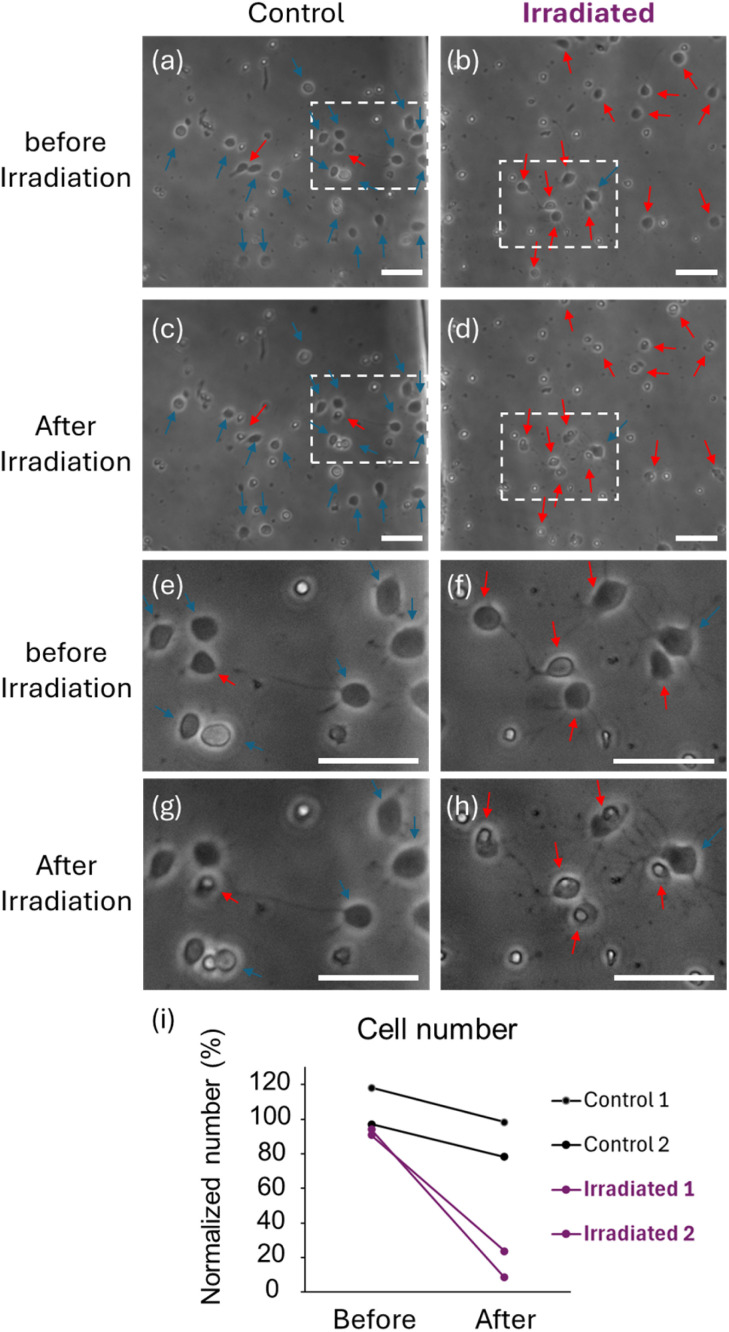
Induction of cell death by focal irradiation of X-rays to one of the two chambers of the CPMS. (a and b) Cells in the chamber before X-ray irradiation. (c) Cells in the chamber 6 h after focal irradiation of X-rays to the chamber shielded by lead plates. The position in the chamber of the CPMS used for image acquisition in (c) was the same as that in (a). (d) Cells in the chamber 6 h after focal irradiation of X-rays without shielding with the lead plate. The position in the chamber for image acquisition in (d) was the same as that in (b). (e, f, g and h) Enlarged images of hippocampal cells in the squares of the images in (a, b, c and d), respectively. In the area shielded by the lead plate, most of the cells did not show pyknotic nuclei (blue arrows, white small spots) 6 h after X-ray irradiation. In the unshielded chamber, many cells became pyknotic (red arrows) 6 h after irradiation, indicating preferential induction of cell death in the irradiated chamber. The scale bars indicate 50 µm. (i) The number of hippocampal cells normalized to the average cell number in the fields before irradiation. The cell number in the chamber without shielding was greatly reduced after irradiation (irradiated 1 and 2) compared with that in the chamber with shielding (control 1 and 2). Each circle indicates the normalized number of live cells.

Finally, we examined whether X-rays can selectively irradiate one of the two chambers in the CPMS because spatially-localized irradiation is essential for analyzing secreted substance-mediated RIBE in the CPMS. We have previously shown that irradiation of 0.5 Gy or 1.0 Gy X-rays to 1 DIV cultured neurons induces delayed cell death in the later stages.^31^ In this study, to observe the effects of selective irradiation in a shorter term, we irradiated one of two chambers with a higher dose (2 Gy) of X-rays. As shown in our previous study, cultured hippocampal cells showed immature morphology with short processes before X-ray irradiation 1 d after seeding (Fig. 12(a, b, e and f)).^31^ Six hours after X-ray irradiation, cells with pyknotic nuclei appeared in the chamber of the CPMS, indicating induction of cell death (Fig. 12(d and h)). In contrast, in the chamber shielded by lead plates, only minor changes in the number of pyknotic cells were observed after irradiation (Fig. 12(c and g)). We found that we can induce preferential cell death in the irradiated chamber of the CPMS.

## Discussion

### Closed and pumpless design approach

In this study, we proposed a closed and pumpless MPS, CPMS, to minimize the cell number and culture medium volume required for cell culture. The volume of the required medium for operating fabricated CPMS was 206 µL. Cells could be cultured for 21 d using CPMS with a closed reservoir. However, cells could not survive in an open reservoir. The volume proportion of the extracellular liquid, including blood and lymph, in the human body is lower than 20%. To reproduce the human body conditions in the MPS with an ideal volume proportion of cultured cells and culture medium, the volume of the culture medium should be lower than the volume of cultured cells.^32,33^ This volume proportion was difficult to create even if we used MPS fabricated using microfabrication methods that exhibit advantages in the reduction of MPS volume. Although the inside volume of the CPMS was still not enough to create an equal volume ratio compared to the human body, it achieved the smallest possible volume because CPMS can culture cells by preventing evaporation.

When cells were cultured under gravity-driven flow, cells could not survive for 7 d with opened reservoirs. To get closer to creating this volume proportion, MPS using a gravity-driven flow has been proposed.^33–35^ In the gravity-driven flow system, pumps and microvalves need not be integrated to circulate the medium in the MPS. This allows us to reduce the cost of fabrication and eliminate bubble formation, which interferes with the operation of MPS. In addition, since the gravity-driven flow system has the advantage of reducing the volume, it allowed us to conduct experiments using rare and expensive cells and tissues, such as primary human cells and differentiated cells from human iPS cells, efficiently. However, since the culture medium is circulated by tilting the entire system in the gravity-driven system, the cell culture medium is attached to the walls of the reservoir. The amount of culture medium evaporated by the gravity-driven system becomes larger than that in a static culture in cell culture dishes or 96-well plates because a large area of culture medium can be exposed to air. Thus, in long-term culture using a gravity-driven flow system, excess culture medium has been used for culture. Consequently, the advantages of microfluidic devices, or MPSs, which have the potential to become high-density systems by reducing excess volume, have not been fully utilized. In our experiments, the cultured hippocampal neurons showed extensive neurite outgrowth only in the CPMS with a closed reservoir. In the proposed CPMS, evaporation of the culture medium did not occur once the humidity in the reservoir reached saturation because there was no contact between the culture medium and the outside. Therefore, it was considered that the cells were successfully cultured because the medium did not evaporate, and the pH of the medium did not significantly change. Since the proposed CPMS design could minimize the volume of the culture medium, it is expected that it will contribute to the development of more ideal MPSs, such as organ-on-a-chip, and organs-on-a-chip, which can utilize the advantages of microfluidic devices.

### Fabrication of the CPMS using PDMS

The reservoirs, chambers, and microchannels of the CPMS were fabricated using PDMS, a material consisting of microfluidic devices. Milli and micro scale structures of CPMS made of PDMS were easy to fabricate using soft lithography and there were no liquid leakage. In a passive microfluidic device including the proposed CPMS, the shape of the chamber or microchannel determined the flow rate and shear stress. Application of PDMS for high fabrication precision can reduce the difference between the flow rate estimated from the model and the actual flow rate measured in the fabricated CPMS.

PDMS is suitable for the fabrication of the CPMS. Hippocampal neurons could be cultured for 21 d. PDMS also exhibits advantages for the fabrication of microfluidic devices with gas permeability.^36^ The gas permeability of PDMS minimizes the difference in the concentration of oxygen and CO_2_ between the inside and outside of the CPMS. The long-term survival of neurons for 21 d *in vitro* and accumulation of synaptic proteins along dendrites in the CPMS support this prediction.

Meanwhile, the permeability of water vapor is a concern in the fabrication of CPMS using PDMS. The surface of PDMS is normally hydrophobic and does not permeate water. However, it has high permeability to water vapor. Bian *et al.* evaluated the permeability of water vapor in PDMS sheets, the thickness of which was from 8 µm to 160 µm.^37^ The vapor transmission rate was inversely correlated to the thickness of the PDMS. The minimum thickness of PDMS in CPMS was over 2 mm, which is the distance to the side of the CPMS. Even though there are no clear data about the permeability of over 2 mm PDMS, it is expected that there is no considerable effect from the permeability of water vapor in CPMS.

The non-specific absorption of proteins and other molecules, such as drugs, on the surface of PDMS has been another concern in the fabrication of CPMS using PDMS. Absorption of proteins and other molecules on the surface of PDMS may reduce their concentration in the cell culture medium in the CPMS.^38^ Coating with parylene can prevent the absorption of proteins and other molecules on the surface of PDMS.^39^ In future work, along with balancing gas permeability, prevention of molecular absorption to PDMS by parylene coating will help us reproduce intercellular communications, such as RIBEs, in the CPMS.

### Unidirectional flow and shear stress

In this study, fluid analysis was performed using the finite element method (FEM) using models with chamber/microchannel height, *h*, of 20 to 500 µm. Chambers/microchannels with heights, *h*, from 20 to 500 µm can be fabricated by soft lithography using PDMS. There was a proportional relationship between the microchannel height and flow rate. The shear stress peaked at a height *h* of 380 µm. The minimum shear stress was 2.2 mN m^−2^ at a height *h* of 20 µm, and the maximum shear stress was 10.2 mN m^−2^ at a height *h* of 380 µm. This is associated with changes in the middle point of the flow field in proportion to the chamber/microchannel height *h*. As the cross-sectional area of the microchannel increased, the volumetric flow rate increased nonlinearly because of reduced hydraulic resistance. However, since the inlet pressure remains constant, the average flow velocity did not change significantly. Additionally, the flow velocity gradient at the wall of the microchannel becomes gentler. Therefore, the shear stress reached an extremum even flow rate increases constantly. Also, these results indicate that the flow rate and the shear stress on the cells can be optimized by changing the chamber/microchannel height.

In the human body, shear stress is approximately 1000 to 7000 mN m^−2^ in arteries and 100 to 600 mN m^−2^ in veins.^40^ The chamber/microchannel height is 404.4 ± 22.9 µm. FEM analysis showed that the neurons suffered under a maximum shear stress of 10.2 mN m^−2^. Since only the vascular endothelial cells and ependymal cells directly sense the fluid shear stress in the human body, the shear stress on neurons and glial cells should be as low as possible. Previous studies have shown that shear stress should be lower than 25 mN m^−2^ when it is applied directly to culture cells.^41^ Therefore, the shear stress applied to cultured neurons in the CPMS was sufficiently small to maintain neuronal survival and growth. On the other hand, the minute flow in the interstitial space of the brain plays a significant role in neuronal differentiation and morphological determination.^42–44^ As shown in the FEM analysis results in Fig. 5 and 6, the CPMS enables control of the shear stress applied to cells by adjusting only the microchannel height while maintaining a constant total culture medium volume. This shows that the CPMS is a platform for strictly assessing the effects of mechanical stimulation on cells. Although the extent to which the microfluidic flow generated by the CPMS affects neurons has not yet been evaluated, the device was shown to be capable of creating a closed and stable microenvironment with a flow, as shown in Fig. 7 and 8. Therefore, it is expected that this system can not only reproduce an environment similar to that of the brain extracellular space for cultured neurons but also a platform to simulate or analyze the effect of mechanical stimulation by small shear stress to various cells in future work.

When 206 µL of fluorescent bead suspension was introduced and the CPMS was tilted at a 9° angle of inclination, the fluorescent beads flowed at a maximum flow rate of 15.7 µL min^−1^. As the water head pressure gradually decreased, the flow rate correspondingly declined. However, since the flow rate remained consistently positive, it was demonstrated that the flow within the device proceeded in a unidirectional flow. Furthermore, when the uranine solution was dropped into one chamber and the CPMS was tilted five times, the fluorescence intensity in the two chambers became comparable in 5 min. These results indicate that the CPMS can generate a one-way flow field in a closed space by tilting the CPMS, even when the chambers were isolated from the outside, and mixing the culture medium between different chambers within 5 min. Additionally, these results indicate an efficient inter-chamber exchange of substances within a few minutes. In MPS and organs-on-a-chip research, devices using unidirectional flow have been developed using pumps to rapidly and accurately reproduce the intercellular communications between cells cultured in different chambers.^45^ Although it is difficult to compare the efficiency of mixing the medium and circulating function between different devices because the conditions of each device, such as size, volume, and the kind of culturing cells differ, we expect that the proposed CPMS will circulate secreted molecules from the cells as or more efficiently than previous devices using unidirectional flow.

### System performance

In the CPMS with closed reservoirs, cultured neurons survived for 21 d, extended neurites, and formed synapses in a static condition. On the other hand, in the CPMS with open reservoirs, most hippocampal cells that survived for 21 d did not have neurites. In a previous study for reducing culture medium by using MPS, 80 µL of culture medium was enough for cells to survive for 72 h.^18^ In the present study, cells were cultured with 206–360 µL of culture medium. Without a top part for closing reservoirs, long-term culture for 21 d seemed to increase evaporation of the culture medium even in a static condition and reduced neuronal survival and neurites, probably by increasing the osmotic pressure and concentration of molecules in the culture medium. In the CPMS with closed reservoirs, the evaporation of the culture medium was sufficiently reduced to maintain long-term neuronal culture for 21 d.

When the culture medium was circulated by a gravity-driven flow, cells cultured for 7 d in the CPMS with open chambers exhibited pyknotic nuclei and did not have neurites. This is probably because the rocking mixer-induced inclination of the CPMS makes the culture medium adhere to the walls of the reservoirs and accelerates the evaporation of the culture medium. Meanwhile, neurons cultured for 7 d in the CPMS with closed reservoirs maintained neurites even when the culture medium was circulated by a gravity-driven flow. These results indicate that our CPMS design worked well to prevent the evaporation of the culture medium and maintain neuronal development with a circulation of 206–360 µL of culture medium. The recently developed devices using a gravity-driven unidirectional flow required approximately 500 µL of culture medium to operate.^46^ Although the purpose of the research was different, our proposed CPMS can be operated with a smaller volume of culture medium compared with the recently developed device and other conventional MPSs. This characteristic of our proposed CPMS can be useful when only a limited number of cells are available (*e.g.*, expensive, commercially available human cells with a limited cell number) and/or intercellular communication mediated by low amounts of secreted molecules from cells needs to be recapitulated (*e.g.*, RIBE).

In this study, immunocytochemical analysis was performed to validate the development and maturation of the neuronal culture in the CPMS using antibodies against a microtubule-associated protein, MAP2, and an actin-binding protein, drebrin. Since the top frame is removable from the CPMS during cell culture, cells in the chambers could be easily chemically fixed for immunocytochemical analysis. This feature allows us to observe fluorescent signals from the neurons after immunocytochemistry, indicating that detailed analysis of the cultured cells in the CPMS is possible.

Focal irradiation of X-rays caused acute apoptosis, mainly in one of the culture chambers in the CPMS. Tilting the CPMS by rocking the mixer could induce the circulation of the culture medium between the two chambers. Since we could conduct focal irradiation only in one side cell culture chamber with neurons and unidirectional flow between chambers, it is expected that the intercellular communication between stimulated and unstimulated cells can be evaluated. Therefore, this system will be useful for the analysis of intercellular communications, such as RIBEs, mediated by circulating substances secreted from the irradiated cells.

## Conclusions

In this study, we proposed a CPMS capable of co-culturing rare cells and focal irradiation with X-rays. In the CPMS, gravity-driven unidirectional flow with a maximum flow rate of 15.7 µL min^−1^ was generated. In addition, cells cultured for 21 d in the CPMS formed a neural network. Furthermore, by fabricating the CPMS using PDMS and thin glass substrates with high transparency, focal irradiation of X-rays to cultured neurons in a culture chamber in the CPMS and detailed observation of microstructures such as the localization of synaptic proteins were possible. The proposed CPMS can induce focal changes in the cells cultured in a chamber of the system within a minimized culture space. Therefore, the CPMS will be useful for recapitulating intercellular communications, such as RIBEs, mediated by small amounts of substances secreted by local changes. Furthermore, since the CPMS is smaller than other MPS with unidirectional flow, our system will permit us to use small quantities of rare and/or expensive cells, such as iPS cell-derived cells and primary human cells, more efficiently.

## Author contributions

Hidetaka Ueno: writing – original draft, conceptualization, methodology, investigation, validation, writing – review & editing, funding acquisition, project administration. Kenji Hanamura: writing – original draft, conceptualization, methodology, investigation, validation, writing – review & editing, funding acquisition, project administration. Yuri Aoki: methodology, investigation, validation. Mai Yamamura: methodology, investigation, validation. Tomoaki Shirao: conceptualization, project administration. Takaaki Suzuki: writing – original draft, conceptualization, methodology, investigation, validation, writing – review & editing, funding acquisition, project administration.

## Conflicts of interest

The author, Tomoaki Shirao is the founder and CEO of AlzMed, Inc. The other authors declare that they have no known competing financial interests or personal relationships that could have appeared to influence the work reported in this paper.

## Data Availability

All data will be made available on request.
